# Maxillary Sinus Dimensions in Skeletal Class I Chinese Population with Different Vertical Skeletal Patterns: A Cone-Beam Computed Tomography Study

**DOI:** 10.3390/diagnostics12123144

**Published:** 2022-12-13

**Authors:** Jingyi Wang, Min Zou, Abby Syverson, Zhong Zheng, Chenshuang Li

**Affiliations:** 1 Department of Orthodontics, School of Dental Medicine, University of Pennsylvania, Philadelphia, PA 19104, USA; 2 Key Laboratory of Shannxi Province for Craniofacial Precision Medicine Research, College of Stomatology, Xi’an Jiaotong University, 98 XiWu Road, Xi’an 710004, China; 3 Clinical Research Center of Shannxi Province for Dental and Maxillofacial Diseases, College of Stomatology, Xi’an Jiaotong University, 98 XiWu Road, Xi’an 710004, China; 4 Department of Orthodontics, College of Stomatology, Xi’an Jiaotong University, 98 XiWu Road, Xi’an 710004, China; 5 David Geffen School of Medicine, University of California, Los Angeles, CA 90095, USA

**Keywords:** maxillary sinus, skeletal vertical pattern, alveolar bone, sex as biological variable (SABV)

## Abstract

Due to the close relationship between the maxillary posterior teeth roots and the maxillary sinus floor, the maxillary sinus can significantly impact various dental treatments, including endodontic procedures and surgical apicectomy, periodontal flap surgery, surgical tooth extraction, dental implantation, and orthognathic surgeries. Specifically, in orthodontics, the location of the maxillary sinus floor may affect tooth movement and insertion of temporary anchorage devices (TADs). This study aims to evaluate the dimensions and location of the maxillary sinus in the Chinese orthodontic patient population with skeletal class I. Using cone-beam computed tomography (CBCT), the volumetric size, height, width, and depth of the sinus and the amount of alveolar bone below the sinus floor and buccal/palatal to the sinus wall were compared between patients of different genders and different vertical skeletal patterns. Unlike the previously reported skeletal class II population, the skeletal class I patients with different vertical patterns do not have significantly different size sinuses. On the other hand, males have larger maxillary sinuses in all parameters than females in the testing population. In addition, no significant correlation was noticed between the SN-MP angle and sinus dimensions or between the ANB angle and sinus dimensions. Nevertheless, the distance from the sinus floor to the alveolar bone crest is not correlated with skeletally sagittal or vertical parameters in females but negatively correlated with the skeletal sagittal parameter in males. In summary, different from the skeletal class II population, there is no significant difference in maxillary sinus size and location among different vertical skeletal patterns in the skeletal class I population. Compared to the skeletal class II population, a higher percentage of the skeletal class I population has an alveolar bone with less than 5 mm thickness, representing a narrowed safe zone of TADs placements.

## 1. Introduction

The maxillary sinus has a pyramidal shape and is the largest paranasal sinus [1]. The anterior wall of the sinus is formed by the facial surface of the maxilla. The posterior wall is formed by the infratemporal surface of the maxilla. The superior wall is formed by the orbit floor. The medial wall separates the sinus from the nasal cavity. The lateral apex of the sinus extends into the zygomatic process of the maxilla. The floor of the sinus is formed by the alveolar and palatine processes of the maxilla and is below the nasal cavity [2].

Due to its location, the maxillary sinus plays an important role in the field of dentistry. When the maxillary sinus is large, the premolar and molar roots tend to have a close relationship to the maxillary sinus, which can have a significant impact on various dental treatments, including endodontic procedure and surgical apicectomy, periodontal flap surgery, surgical tooth extraction, dental implantation, orthognathic surgeries, or surgical intervention for space-occupying lesions involving the maxillary sinus and the maxilla [3]. In orthodontics, the sinus may affect the biological limitation of tooth movement and the safety area for temporary anchorage devices (TADs) insertion. A previous study has demonstrated that the root contact with the cortical plate at the root apex level during orthodontic tooth movement has been associated with root resorption [4]. As the sinus floor and sinus walls are formed by the cortical bone layer, moving teeth through the maxillary sinus floor mesiodistally or vertically (intrusion) is challenging [3]. It will make the treatment time longer, create periodontal complications, and introduce severe root resorption. On another hand, sinus infection, hematoma, and mucocele have been reported when TADs penetrate the sinus wall [5]. Thus, evaluating the sinus size and position concerning the alveolar ridge is crucial.

Our group recently revealed that, in the skeletal class II population, the skeletal high-angle patients have statistically significantly larger maxillary sinuses than the low- and normal-angle patients in both genders [6], while no significant difference was found in sinus size between genders. Interestingly, it has been reported by other groups that women generally have a smaller sinus volume than men [7,8]. In addition, other groups have different conclusions regarding the correlation between the vertical skeletal patterns and the maxillary sinus size. For example, Endo et al. found that maxillary sinus length, maxillary sinus height, and total maxillary sinus area showed significant positive correlations with upper anterior facial height by evaluating the lateral cephalometric images [9]; Ryu et al. also found that the patients with an anterior open bite have greater maxillary sinus height and more vertical pneumatization of the maxillary sinus floor in the posterior tooth root region than the patients without anterior open bite [10]. On the other hand, Göymen et al. stated there was no difference in maxillary sinus sizes among patients with different vertical patterns [11]; Kosumarl et al. also did not find a significant difference in the distance from the maxillary root apices of posterior teeth to the floor of the maxillary sinus between subjects with a skeletal open bite or skeletal normal bite [12]. However, Oksayan et al. concluded that patients with a low-angle vertical facial pattern have larger maxillary sinus dimensions than patients with a high-angle vertical facial pattern [13]. The disagreements may be due to overlooked sagittal skeletal patterns in the study designs. Nevertheless, several previous publications utilized 2D lateral cephalometric or panoramic images, which introduced systematic error during the measurement due to the overlapping of anatomic structures and built-in magnification in the 2D X-rays [14].

Thus, in the current study, by utilizing previously established protocol [6], we evaluated the size of the maxillary sinus and the amount of alveolar bone around the sinus in Asian skeletal Class I patients with different vertical patterns by utilizing 3D CBCT images, which would help to further understand the anatomic differences for the patients with different skeletal patterns.

## 2. Materials and Methods

### 2.1. Patients

The study was conducted in accordance with the Declaration of Helsinki and approved by the Institutional Review Board of both involved research institutions (protocol # 844317 and date of approval: 20 November 2020; and xjkqll [2020]NO.014 and date of approval: 26 August 2020), with a previously obtained cone-beam computed tomography (CBCT) database of patients seeking orthodontic treatment between 2017 and 2020 [6]. The CBCT images were collected from the orthodontic department, utilizing CBCT imaging as part of the routine pre-orthodontic records exams. Each patient was requested to sit straight and maintain maximal intercuspation of their jaws. All images were taken using (i-Cat, Imaging Sciences International, Hatfield, PA, USA) cone beam machine at 120 kV, 5 mA, 14 cm × 17 cm FOV, 0.4 mm voxel, and scan time of 8.9 s. Six hundred pre-treatment full-volume CBCT images were screened based on the following inclusive and exclusive criteria.

**Figure 1:**
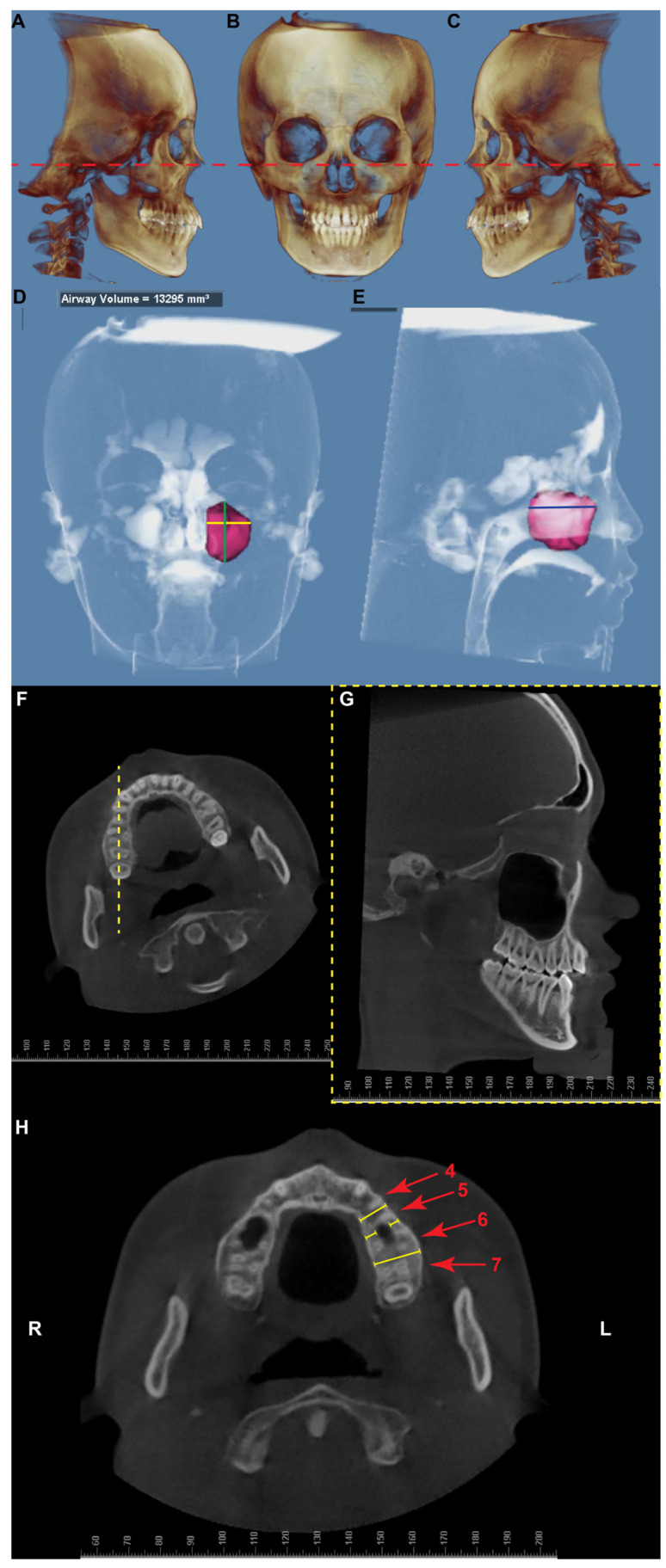
The demography of CBCT measurement protocol used in the current study. (**A**–**C**) The CBCT image was oriented to Frankfort horizontal plane (red dash line) using right and left porions (**A**, front view) and the right orbitale (**B**, right view), then verified on the left view (**C**). (**D**) Dolphin Software 3D airway/sinus rendering module was utilized to measure the volume of the maxillary sinuses. The sinus volume was directly reported by the software. The image showed the left maxillary sinus of this patient. The height (solid green line) and width (solid yellow line) of the maxillary sinus were measured on the front view. (**E**) The depth of the maxillary sinus (solid blue line) was measured on the side view. (**F**) The CBCT image was oriented to have the sagittal cut (yellow dash line) midway between the buccal and palatal cortices for the alveolar bone height measurement between every two posterior teeth. (**G**) The sagittal view generated from the orientation shown in panel F for the alveolar bone height measurement between every two posterior teeth. (**H**) The demography of alveolar bone thickness measurement buccally and palatally to the maxillary sinus on the left side of the patient. An axial slice of a CBCT image at the level apical to the maxillary posterior alveolar bony crest. The red numbers and arrows indicate the roots of the posterior teeth—4, first premolar; 5, second premolar; 6, first molar; 7, second molar. The alveolar bone thickness measurements on this slice were marked as yellow lines. Between the right first and second premolars, and between the first and second molars, no maxillary sinus penetration was noticed. Thus, the measurements were made from the buccal cortex to the palatal cortex in this location to represent the alveolar bone thickness—R, right side; L, left side.

The initial screening inclusive criteria were as follows: (1) 15–40 years by age; (2) permanent dentition with the second molar fully erupted; (3) skeletal class I with ANB angle between 0.7° and 4.7° based on Chinese cephalometric norm as all the CBCT images were obtained on Chinese patients [15,16,17]. The initial screening exclusive criteria were as follows: (1) craniofacial syndromes; (2) obvious deformity of the maxilla, including asymmetry; (3) the previous history of orthodontic treatment; (4) the previous history of craniofacial trauma; and (5) history of severe periodontal diseases. The voxel size of all the images was 0.400 mm × 0.400 mm × 0.400 mm. After initial screening, 157 CBCT images were imported into the Dolphin 3D software (Dolphin Imaging; version 11.95 Premium, Chatsworth, CA, USA). Then, the image was oriented to Frankfort horizontal plane using right and left porions and the right orbitale (Figure 1A–C), and screened to exclude the images with posterior periapical defects, posterior impacted/missing teeth (except for third molars), posterior teeth with root canal treatment, maxillary sinus pathology, or supernumerary teeth. After the second round of screening, patients were cataloged into low-, normo-, and high-angle groups based on the Chinese norm of SN-MP angle: <27.3° as low-angle, 27.3–37.7° as normo-angle, and >37.7° as high-angle [15,17]. Both left and right sides were evaluated, and if any potential radiological abnormality was detected, that side was excluded for further analyses.

### 2.2. Sample Size Calculation

As stated in the previous study [6], 12 sites (minimum 6 patients, including left and right sides) are needed based on power analysis with α = 0.05, 80% power, and a Cohen’s *d* of 1.2 that representing a ‘very large’ effect size [18] to ensure an adequate sample size for showing statistical differences.

### 2.3. Maxillary Sinus Size and Location Measurements

The measurement procedure completely follows the previously well-established protocol [6]. In brief, Dolphin Software 3D airway/sinus rendering module was utilized to measure the volume of the maxillary sinuses (Figure 1D–E). Particularly, the height (from the most superior point to the most inferior point), width (from the most medial point to the most lateral point), and depth (from the most anterior point to the most posterior point) of each maxillary sinus were measured in a 2D plane on the 3D reconstructed sinus image.

The CBCT image was oriented to have the sagittal cut midway between the buccal and palatal cortices for the alveolar bone height measurement between every two posterior teeth (Figure 1F). The alveolar bone height was defined as the distance between the lowest point of the cortical boundary of the sinus floor and the alveolar bone crest (Figure 1G). The measuring lines were perpendicular to the occlusal surface of the adjacent teeth.

Lastly, the width of the alveolar bone between the maxillary sinus wall and the buccal/palatal cortices of the alveolar ridge was measured at the levels of 5, 8, and 10 mm apically from the alveolar bone crest. If the sinus was not present at a certain level of the alveolus, then the whole thickness of the alveolar ridge was recorded as the measurements for both the buccal and the palatal sides (Figure 1H).

### 2.4. Statistical Analysis

All the measurements were taken by the same examiner (J.W.). To confirm the intra-examiner reliability of the current protocol, six CBCT files were randomly selected and measured twice with an interval of at least one month, and the interclass correlation coefficient (ICC) was assessed by the IBM SPSS software (Statistical Package for Social Sciences version 26.0, Chicago, IL, USA). A Shapiro–Wilk normality test was conducted by OriginPro 8 (Origin Lab Corp., Northampton, MA, USA), revealing that some data did not follow the normal distribution. Therefore, data are presented with a median [minimum, maximum]. In addition, a Mann–Whitney *U* test for statistical comparison and Pearson’s correlation coefficient (*r*) calculation were also performed using OriginPro 8.

## 3. Results

### 3.1. Patient Demographic Information

After CBCT image screening as described above, the final patient count and side count are summarized in Table 1, demonstrating that the power analysis determined minimum required sample size (N = 12/group) is met.

No statistically significant difference in the age and ANB angle was detected among the included low-, normo-, and high-angle skeletal class I patients (Table 1). On the contrary, the SN-MP angles were remarkedly distinguished among included patients with different basic malocclusion classifications (Table 1).

The ICC for the repeated measurements of the six randomly selected CBCT images was 0.969. This high intra-examiner ICC is consistent with the previous study that assessed the class II population (0.976) [6], supporting the high consistency and reliability of the current CBCT-based measurement protocol regardless of the basic malocclusion classification of patients.

### 3.2. Comparison of Maxillary Sinus Size

In the skeletal class II, high-angle subjects had a statistically larger sinus volume than normo- and low-angle subjects in both genders [6], while in the current study, no difference was detected among different vertical groups in the skeletal class I (Table 2). The same trends were also noticed in the measurements of maxillary sinus height (Table 2), width (Table 2), and depth (Table 2).

Concerning sex as a biological variable (SABV), female skeletal class II subjects had a larger maxillary sinus width than their male counterparts [6]. On the contrary, skeletal class I female subjects had a smaller maxillary sinus than male subjects regarding all four measured parameters (Table 2).

In the skeletal class II population, a positive and statistically significant correlation was detected between the SN-MP angle and all dimensional sinus measurements in both genders, and a positive correlation between ANB angle and sinus size in female subjects [6]. However, a statistically meaningful correlation was neither observed between the SN-MP angle and sinus size nor between the ANB angle and sinus size in the skeletal class I population (Table 3).

### 3.3. Maxillary Sinus Floor to the Alveolar Bone Crest

We further evaluate the distance from the maxillary sinus floor to the alveolar bone crest between each two maxillary posterior teeth. Generally, the alveolar bone height between the first and second premolars (4 and 5) was greater than that between the second premolar and first molar (5 and 6) and between the first and second molar (6 and 7) (Table 4). When considering SABV, no statistically significant difference was detected, neither in the comparison among vertical patterns at each location. This differs from the skeletal class II population in which the high-angle subjects had a lower alveolar bone height than their low-angle counterparts [6].

No correlation with statistical significance was detected, with one exception: a negative correlation between the ANB angle and the distance from the maxillary sinus floor to the alveolar bone crest at all three evaluated locations was detected in male subjects; however, this negative correlation was not replicated in the female subjects (Table 3).

### 3.4. Maxillary Sinus Walls to the Buccal and Palatal Alveolar Bone Cortices at Different Vertical Levels

At the level of 5 mm above the alveolar bone crest (Table 5), in general, the alveolar ridge became wider in the posterior than in the anterior part of the assessed skeletal class I patients, which is similar to the trend observed in the skeletal class II population previously [6]. In addition, similar to the skeletal class II population, males tend to have a wider alveolar ridge than females in the skeletal class I population (Table 5). At this level, no sinus penetration was detected in all six groups at the location between 4 and 5, and no statistical significance was observed in the different vertical patterns of all buccal and palatal measurements.

At the level of 8 mm above the alveolar bone crest (Table 6), differences in the skeletal class I patients were only found in the location between first and second premolars, where a wider alveolar ridge was likely observed in males than females and where the high-angle subjects tended to have a narrower alveolar bone thickness than the low-angle subjects regardless the gender.

We further evaluated superiorly at the level of 10 mm above the alveolar bone crest (Table 7). Similar to the level of 8 mm, the high-angle skeletal class I subjects tended to have a thinner alveolar bone between the first and second premolars than the low-angle subjects without discrimination in genders. On the other hand, the skeletal class I males tend to have a wider alveolar ridge than females between the first and second premolars, especially in the high-angle subpopulation.

Lastly, we checked the incidence of alveolar bone thickness less than 5 mm in all the evaluated locations, as 5 mm is the general depth of TAD insertion [19]. Similar to the trends observed in the skeletal class II population [6], the odds of alveolar bone with less than 5 mm thickness increased from anterior to posterior (Table 8). Meanwhile, in the males, the high-angle group has a higher incidence of less than 5-mm-thick alveolar bone, while a similar trend is not observed in females.

## 4. Discussion

Several studies have been done to relate the maxillary sinus size to the sagittal and vertical skeletal patterns, while controversial conclusions were reached in these pioneered investigations. For example, from the sagittal perspective, Oktay et al. concluded that females with class II have relatively larger sinuses [20], while Endo et al. did not find the same tendency [9]; and regarding the vertical patterns, Ryu et al. found that there was greater sinus height and less basal bone in subjects with open bite [10]; however, Kosumarl et al. concluded that there was no difference in sinus height between subjects with a skeletal open bite and those with normal open bite [12]. The discrepancy could be attributed to multiple limitations, such as only using 2D imaging, small sample size, and, more importantly, not always having clear skeletal classification in both vertical and sagittal dimensions.

By only focusing on the skeletal class II population, our previous study demonstrated a strong correlation between the vertical skeletal pattern and the maxillary sinus size and dimension, as well as a correlation to the amount of alveolar bone around the sinus [6]. Moreover, we also showed that gender is an important contributor to determining the sinus size and dimension and the surrounding alveolar bone of the skeletal class II population [6]. Considering the majority of the previous reports described some differences in sinus dimensions between skeletal class I and class II subjects [20], in the current study, we extend our previous evaluation to the skeletal class I population.

Surprisingly, we found no significant difference in the maxillary sinus size within different vertical skeletal patterns in the class I population, further highlighting the importance of the sagittal skeletal pattern in evaluating the relationship between maxillary sinus size and the vertical skeletal patterns, and may explain the variation on the conclusions among previous studies, as a mixture of subjects with different sagittal skeletal patterns may be included in each vertical pattern group. In addition, more gender-related differences in maxillary sinus size were observed in the skeletal class I population, which is distinct from the skeletal class II subjects.

When evaluating the amount of alveolar bone around the maxillary sinus in these skeletal class I subjects, again, unlike the skeletal class II population, no significant difference was found regarding the distance between the alveolar bone crest to the sinus floor within different vertical skeletal patterns, nor on the alveolar bone thickness at the level of 5 mm from the alveolar bone crest. In addition, differences were noticed in the superior levels at 8 mm and 10 mm at the premolar regions but not posterior in the skeletal class I population. These observations are also different from those observed in the skeletal class II population, in which vertical skeletal pattern-associated differences were in the molar region instead of the anterior region.

Among the skeletal class I subjects evaluated in the current study, the different incidence of alveolar bone less than 5 mm thick in relation to skeletal vertical patterns was only observed in males but not females. However, the incidence of alveolar bone with a thickness less than 5 mm in female subjects with high-angle is lower in the skeletal class I population than the skeletal class II counterparts. Moreover, compared to the molar areas, the premolar region remains a relatively safe region for TADs placement for both males and females in the skeletal class I population, similar to the previous observation concerning the skeletal class II patterns [6].

There are certain limitations of the current study that need to be taken into consideration. Firstly, the current study involved subjects from 15 to 40 years old. Most maxillary sinus postnatal growth happens during the first three years of life and between 7 and 12 years of age. The adult sinus size is usually reached between 12 and 15 years of age, and its development overlaps with the peak of growth in both males and females. After 18 years of age, the maxillary sinus volume usually decreases with age [7,8]. Therefore, we picked the age range representing the matured size of the maxillary. However, the sinus size and the distance between the sinus floor and alveolar crest are dynamically changing during aging or when there is posterior tooth loss [21,22]. Thus, the findings from the current study may not be applied to the patient with an age out of the age range used in this study. Secondly, only Chinese subjects were evaluated in the current study. Differences in craniofacial anatomy among racial groups have been documented in a variety of structures, but the oral and maxillofacial regions are particularly defining regions of variability between different racial/ethnic groups. In fact, analysis of the maxillary sinus has been suggested to be used for ethnic group identification of a cranium of unknown origin [23], which highlights race-related differences in the maxillary sinus. Thus, the findings from the current study may not be applied to patients with other ethnic backgrounds.

## 5. Conclusions

Unlike the skeletal class II population, there was no significant difference in the maxillary sinus size within different vertical skeletal patterns in the skeletal class I population. However, males tend to have larger maxillary sinuses than females. In addition, in the class I population, the maxillary sinus perforation risk during TADS insertion is not significantly different regarding different vertical groups in females; however, in males, it differs among different vertical patterns with an increase from low to normal to high-angle. Compared to the skeletal class II population, a higher percentage of the skeletal class I population has an alveolar bone with less than 5 mm thickness, representing a narrowed safe zone of TADs placements. Although a general trend was found in the current study, the large data variation within groups for each measurement further emphasizes the importance of personalized evaluation during clinical dentistry diagnosis and treatment planning.

## Figures and Tables

**Table 1:** The demographic information of the subjects included in the current study. L: low angle; N: normal angle; H: high angle.

	Patient Count	Side Count	Age (Years) Median [Min, Max]	ANB Angle (Degrees) Median [Min, Max]	SN-MP Angle Median [Min, Max]
Female L	11	21	20.40 [18.7, 29.3]	2.50 [1.1, 4.4]	24.40 [20.8, 26.6]
Female N	11	21	20.90 [16.1, 35.1]	3.20 [0.7, 4.2]	34.30 [31.1, 37.2]
Female H	10	20	22.50 [18.0, 28.7]	3.00 [0.8, 4.4]	38.30 [37.9, 43.6]
Male L	10	18	19.50 [15.0, 25.7]	2.45 [0.8, 4.7]	23.35 [19.3, 26.8]
Male N	11	21	20.60 [15.2, 29.5]	3.00 [0.7, 4.3]	31.50 [28.6, 37.4]
Male H	7	14	16.70 [15.5, 40.7]	2.90 [1.0, 4.5]	40.10 [38.2, 44.7]

**Table 2:** The comparison of maxillary sinus size among groups. L: low angle; N: normal angle; H: high angle. *: *p* < 0.05.

Parameter	Group	Median [Min, Max]	*p*-Value of Mann–Whitney *U* Test					
			Compare to Female L	Compare to Female N	Compare to Female H	Compare to Male L	Compare to Male N	Compare to Male H
Sinus Volume (mm^3^)	Female L	15719 [6639, 25419]	-	0.0257 *	0.3318	0.0431 *	-	-
	Female N	13628 [6746, 20944]	0.0257 *	-	0.3449	-	0.0028 *	-
	Female H	15249 [6936, 21425]	0.3318	0.3449	-	-	-	0.0062 *
	Male L	21211 [7731, 24129]	0.0431 *	-	-	-	0.3945	0.4643
	Male N	18543 [7796, 25393]	-	0.0028 *	-	0.3945	-	0.1976
	Male H	21535 [10817, 33139]	-	-	0.0062 *	0.4643	0.1976	-
Sinus Height (mm)	Female L	36.40 [27.90, 40.60]	-	0.1661	0.6468	0.0094 *	-	-
	Female N	34.80 [15.20, 40.20]	0.1661	-	0.1790	-	0.0001 *	-
	Female H	37.15 [29.30, 44.00]	0.6468	0.1790	-	-	-	0.0005 *
	Male L	40.25 [21.60, 45.80]	0.0094 *	-	-	-	0.7120	0.1535
	Male N	42.00 [27.70, 45.50]	-	0.0001 *	-	0.7120	-	0.1468
	Male H	43.90 [34.20, 52.50]	-	-	0.0005 *	0.1535	0.1468	-
Sinus Width (mm)	Female L	27.80 [20.20, 37.30]	-	0.0837	0.5567	0.3244	-	-
	Female N	26.10 [12.70, 30.90]	0.0837	-	0.3897	-	0.0205 *	-
	Female H	27.25 [19.40, 30.40]	0.5567	0.3897	-	-	-	0.0230 *
	Male L	29.45 [20.60, 35.00]	0.3244	-	-	-	0.8838	0.3423
	Male N	28.60 [19.40, 34.20]	-	0.0205 *	-	0.8838	-	0.2667
	Male H	31.30 [23.00, 37.40]	-	-	0.0230 *	0.3423	0.2667	-
Sinus Depth (mm)	Female L	34.70 [30.60, 41.40]	-	0.0630	0.3826	0.0502	-	-
	Female N	34.00 [22.60, 38.50]	0.063	-	0.4975	-	0.0066 *	-
	Female H	34.75 [25.80, 40.20]	0.3826	0.4975	-	-	-	0.0047 *
	Male L	37.95 [29.40, 40.70]	0.0502	-	-	-	0.2783	0.3718
	Male N	36.30 [31.40, 44.40]	-	0.0066 *	-	0.2783	-	0.1123
	Male H	37.75 [30.60, 46.30]	-	-	0.0047 *	0.3718	0.1123	-

**Table 3:** Pearson’s correlation coefficient (*r*) calculation and statistical analysis results between maxillary sinus measurements and SN-MP angle or ANB angle for all included skeletal class I subjects. 4: first premolar, 5: second premolar, 6: first molar, 7: second molar. *: *p* < 0.05.

	Parameter	Female		Male	
		*r*	*p*-Value	*r*	*p*-Value
SN-MP angle	Sinus volume	−0.2088	0.1034	0.0131	0.9257
	Sinus height	0.0023	0.9860	0.1586	0.2566
	Sinus width	−0.2133	0.0961	−0.0897	0.5230
	Sinus depth	−0.1859	0.1479	0.0957	0.4955
	Alveolar bone height between 4 & 5	0.1990	0.1210	−0.0808	0.5654
	Alveolar bone height between 5 & 6	0.2173	0.0898	−0.1045	0.4563
	Alveolar bone height between 6 & 7	0.1068	0.4087	−0.1037	0.4599
ANB angle	Sinus volume	0.0794	0.5397	0.1919	0.1687
	Sinus height	−0.0656	0.6127	0.0924	0.5103
	Sinus width	−0.0901	0.4863	0.1003	0.4749
	Sinus depth	0.0734	0.5708	0.2590	0.0611
	Alveolar bone height between 4 & 5	−0.0416	0.7485	−0.3224	0.0186 *
	Alveolar bone height between 5 & 6	0.0474	0.6131	−0.2775	0.0442 *
	Alveolar bone height between 6 & 7	0.1850	0.1500	−0.3224	0.0186 *

**Table 4:** The comparison of the alveolar bone height from the maxillary sinus floor to the alveolar bone crest among groups. L: low-angle; N: normo-angle; H: high-angle. 4: first premolar, 5: second premolar, 6: first molar, 7: second molar.

Parameter	Group	Median [Min, Max]	*p*-Value of Mann–Whitney *U* Test					
			Compare to Female L	Compare to Female N	Compare to Female H	Compare to Male L	Compare to Male N	Compare to Male H
between 4 & 5 (mm)	Female L	12.40 [7.20, 15.90]	-	0.7889	0.2303	0.0676	-	-
	Female N	12.50 [9.00, 26.80]	0.7889	-	0.3896	-	0.1474	-
	Female H	14.15 [8.00, 19.30]	0.2303	0.3896	-	-	-	0.3274
	Male L	13.60 [10.10, 21.60]	0.0676	-	-	-	0.8618	0.2026
	Male N	14.00 [7.00, 27.00]	-	0.1474	-	0.8618	-	0.1162
	Male H	12.30 [5.80, 22.30]	-	-	0.3274	0.2026	0.1162	-
between 5 & 6 (mm)	Female L	9.50 [4.50, 14.00]	-	0.9059	0.2253	0.2540	-	-
	Female N	9.00 [7.10, 24.00]	0.9059	-	0.3414	-	0.4888	-
	Female H	10.55 [3.70, 16.90]	0.2253	0.3414	-	-	-	0.1061
	Male L	9.75 [5.40, 19.40]	0.2540	-	-	-	0.8291	0.0928
	Male N	10.60 [5.10, 17.20]	-	0.4888	-	0.8291	-	0.1285
	Male H	8.55 [4.50, 17.20]	-	-	0.1061	0.0928	0.1285	-
between 6 & 7 (mm)	Female L	8.90 [5.00, 10.60]	-	0.8763	0.3752	0.7015	-	-
	Female N	8.60 [5.20, 24.40]	0.8763	-	0.6283	-	0.4067	-
	Female H	9.25 [4.10, 12.80]	0.3752	0.6283	-	-	-	0.2939
	Male L	8.90 [6.90, 15.50]	0.7015	-	-	-	0.7329	0.3051
	Male N	9.60 [4.60, 16.50]	-	0.4067	-	0.7329	-	0.2064
	Male H	8.40 [3.60, 12.60]	-	-	0.2939	0.3051	0.2064	-

**Table 5:** The comparison of buccal and palatal alveolar bone thickness among groups at the level 5 mm above the alveolar crest. L: low-angle; N: normo-angle; H: high-angle. 4: first premolar, 5: second premolar, 6: first molar, 7: second molar. *: *p* < 0.05.

Parameter		Group	Median [Min, Max]	*p*-Value of Mann–Whitney *U* Test					
				Compare to Female L	Compare to Female N	Compare to Female H	Compare to Male L	Compare to Male N	Compare to Male H
Buccal	between 4 & 5 (mm)	Female L	9.70 [7.60, 11.80]	-	0.4499	0.7714	0.0007 *	-	-
		Female N	9.70 [8.30, 12.40]	0.4499	-	0.2100	-	0.0848	-
		Female H	9.30 [7.50, 11.10]	0.7714	0.2100	-	-	-	0.0094 *
		Male L	10.85 [8.60, 12.40]	0.0007 *	-	-	-	0.3978	0.3416
		Male N	10.70 [8.60, 12.60]	-	0.0848	-	0.3978	-	0.9536
		Male H	10.60 [9.20, 12.10]	-	-	0.0094 *	0.3416	0.9536	-
	between 5 & 6 (mm)	Female L	11.60 [3.00, 14.60]	-	0.3023	0.6840	0.0051 *	-	-
		Female N	11.70 [9.30, 14.50]	0.3023	-	0.4890	-	0.0707	-
		Female H	11.70 [2.30, 12.80]	0.684	0.4890	-	-	-	0.0179 *
		Male L	12.30 [10.90, 14.50]	0.0051 *	-	-	-	0.8399	0.9477
		Male N	12.60 [1.50, 15.80]	-	0.0707	-	0.8399	-	0.9801
		Male H	12.50 [11.20, 15.20]	-	-	0.0179 *	0.9477	0.9801	-
	between 6 & 7 (mm)	Female L	14.90 [12.60, 16.50]	-	0.0788	0.2096	0.0082 *	-	-
		Female N	14.30 [12.10, 16.60]	0.0788	-	0.6556	-	0.0005 *	-
		Female H	14.15 [13.50, 15.90]	0.2096	0.6556	-	-	-	0.6222
		Male L	15.95 [13.30, 17.80]	0.0082 *	-	-	-	0.9389	0.2961
		Male N	15.70 [1.70, 18.20]	-	0.0005 *	-	0.9389	-	0.3724
		Male H	14.75 [1.30, 17.90]	-	-	0.6222	0.2961	0.3724	-
Palatal	between 4 & 5 (mm)	Female L	9.70 [7.60, 11.80]	-	0.4499	0.7714	0.0007 *	-	-
		Female N	9.70 [8.30, 12.40]	0.4499	-	0.2100	-	0.0848	-
		Female H	9.30 [7.50, 11.10]	0.7714	0.2100	-	-	-	0.0094 *
		Male L	10.85 [8.60, 12.40]	0.0007 *	-	-	-	0.3978	0.3416
		Male N	10.70 [8.60, 12.60]	-	0.0848	-	0.3978	-	0.9536
		Male H	10.60 [9.20, 12.10]	-	-	0.0094 *	0.3416	0.9536	-
	between 5 & 6 (mm)	Female L	11.60 [3.70, 14.60]	-	0.3023	0.6840	0.0051 *	-	-
		Female N	11.70 [9.30, 14.50]	0.3023	-	0.4890	-	0.0707	-
		Female H	11.70 [2.40, 12.80]	0.6840	0.4890	-	-	-	0.0179 *
		Male L	12.30 [10.90, 14.50]	0.0051 *	-	-	-	0.8399	0.9477
		Male N	12.60 [2.20, 15.80]	-	0.0707	-	0.8399	-	0.9801
		Male H	12.50 [11.20, 15.20]	-	-	0.0179 *	0.9477	0.9801	-
	between 6 & 7 (mm)	Female L	14.90 [12.60, 16.50]	-	0.0788	0.2096	0.0082 *	-	-
		Female N	14.30 [12.10, 16.60]	0.0788	-	0.6556	-	0.0005 *	-
		Female H	14.15 [13.50, 15.90]	0.2096	0.6556	-	-	-	0.6222
		Male L	15.95 [13.30, 17.80]	0.0082 *	-	-	-	0.9389	0.2961
		Male N	15.70 [3.90, 18.20]	-	0.0005 *	-	0.9389	-	0.3724
		Male H	14.75 [3.10, 17.90]	-	-	0.6222	0.2961	0.3724	-

**Table 6:** The comparison of buccal and palatal alveolar bone thickness among groups at the level 8 mm above the alveolar crest. L: low-angle; N: normo-angle; H: high-angle. 4: first premolar, 5: second premolar, 6: first molar, 7: second molar. *: *p* < 0.05.

Parameter		Group	Median [Min, Max]	*p*-Value of Mann–Whitney *U* test					
				Compare to Female L	Compare to Female N	Compare to Female H	Compare to Male L	Compare to Male N	Compare to Male H
Buccal	between 4 & 5 (mm)	Female L	10.10 [7.60, 14.80]	-	0.6948	0.0006 *	0.0098 *	-	-
		Female N	10.00 [7.00, 13.00]	0.6948	-	0.0014 *	-	0.8763	-
		Female H	8.55 [6.10, 11.00]	0.0006 *	0.0014 *	-	-	-	0.0266 *
		Male L	11.50 [8.80, 13.10]	0.0098 *	-	-	-	0.0037 *	0.0097 *
		Male N	10.50 [2.00, 14.20]	-	0.8763	-	0.0037 *	-	0.8877
		Male H	9.90 [1.60, 12.70]	-	-	0.0266 *	0.0097 *	0.8877	-
	between 5 & 6 (mm)	Female L	11.50 [1.10, 14.90]	-	0.8960	0.3030	0.3385	-	-
		Female N	12.20 [2.40, 16.20]	0.8960	-	0.1663	-	0.7890	-
		Female H	11.05 [0.80, 13.20]	0.3030	0.1663	-	-	-	0.5982
		Male L	12.75 [2.70, 14.60]	0.3385	-	-	-	0.7433	0.4815
		Male N	11.90 [1.20, 15.60]	-	0.7890	-	0.7433	-	0.7209
		Male H	11.45 [1.20, 15.90]	-	-	0.5982	0.4815	0.7209	-
	between 6 & 7 (mm)	Female L	14.70 [1.10, 16.60]	-	0.5539	0.5831	0.0010 *	-	-
		Female N	14.30 [1.00, 17.00]	0.5539	-	0.8515	-	0.0984	-
		Female H	14.60 [1.50, 16.10]	0.5831	0.8515	-	-	-	0.3630
		Male L	16.10 [5.40, 18.70]	0.0010 *	-	-	-	0.7016	0.4139
		Male N	15.80 [1.10, 18.60]	-	0.0984	-	0.7016	-	0.7332
		Male H	14.80 [1.20, 18.30]	-	-	0.3630	0.4139	0.7332	-
Palatal	between 4 & 5 (mm)	Female L	10.10 [7.60, 14.80]	-	0.6948	0.0006 *	0.0098 *	-	-
		Female N	10.00 [7.00, 13.00]	0.6948	-	0.0014 *	-	0.8763	-
		Female H	8.55 [3.40, 11.00]	0.0006 *	0.0014 *	-	-	-	0.0266 *
		Male L	11.50 [8.80, 13.10]	0.0098 *	-	-	-	0.0037 *	0.0097 *
		Male N	10.50 [2.20, 14.20]	-	0.8763	-	0.0037 *	-	0.8877
		Male H	9.90 [1.60, 12.70]	-	-	0.0266 *	0.0097 *	0.8877	-
	between 5 & 6 (mm)	Female L	11.50 [2.40, 14.90]	-	0.9256	0.2009	0.4385	-	-
		Female N	12.20 [1.60, 16.20]	0.9256	-	0.2056	-	0.6675	-
		Female H	11.05 [1.10, 13.20]	0.2009	0.2056	-	-	-	0.5982
		Male L	12.75 [2.30, 14.60]	0.4385	-	-	-	0.7752	0.4358
		Male N	11.90 [2.20, 15.60]	-	0.6675	-	0.7752	-	0.7085
		Male H	11.45 [2.00, 15.90]	-	-	0.5982	0.4358	0.7085	-
	between 6 & 7 (mm)	Female L	14.70 [4.40, 16.60]	-	0.4734	0.3755	0.0037 *	-	-
		Female N	14.30 [2.20, 17.00]	0.4734	-	0.9948	-	0.1405	-
		Female H	14.60 [3.10, 16.10]	0.3755	0.9948	-	-	-	0.4019
		Male L	16.10 [3.30, 18.70]	0.0037 *	-	-	-	0.8074	0.4139
		Male N	15.80 [1.80, 18.60]	-	0.1405	-	0.8074	-	0.6355
		Male H	14.80 [2.40, 18.30]	-	-	0.4019	0.4139	0.6355	-

**Table 7:** The comparison of buccal and palatal alveolar bone thickness among groups at the level 10 mm above the alveolar crest. L: low-angle; N: normo-angle; H: high-angle. 4: first premolar, 5: second premolar, 6: first molar, 7: second molar. *: *p* < 0.05.

Parameter		Group	Median [Min, Max]	*p*-Value of Mann–Whitney *U* Test					
				Compare to Female L	Compare to Female N	Compare to Female H	Compare to Male L	Compare to Male N	Compare to Male H
Buccal	between 4 & 5 (mm)	Female L	11.10 [1.30, 13.60]	-	0.8960	0.0553	0.0437 *	-	-
		Female N	10.70 [2.00, 15.10]	0.8960	-	0.0036 *	-	0.8665	-
		Female H	8.65 [1.80, 11.10]	0.0553	0.0036 *	-	-	-	0.0180 *
		Male L	11.95 [4.80, 15.30]	0.0437 *	-	-	-	0.0272 *	0.0295 *
		Male N	9.90 [1.80, 13.90]	-	0.8665	-	0.0272 *	-	0.9934
		Male H	10.10 [1.20, 14.30]	-	-	0.0180 *	0.0295 *	0.9934	-
	between 5 & 6 (mm)	Female L	2.90 [1.30, 15,20]	-	0.6764	0.8923	0.0157 *	-	-
		Female N	2.90 [1.20, 16.00]	0.6764	-	0.9846	-	0.3206	-
		Female H	10.50 [0.50, 14.10]	0.8923	0.9846	-	-	-	0.7363
		Male L	13.05 [1.90, 16.30]	0.0157 *	-	-	-	0.2720	0.0663
		Male N	10.60 [1.00, 16.30]	-	0.3206	-	0.2720	-	0.3367
		Male H	2.90 [0.60, 18.40]	-	-	0.7363	0.0663	0.3367	-
	between 6 & 7 (mm)	Female L	3.50 [1.00, 17.10]	-	0.8960	0.4264	0.1072	-	-
		Female N	4.70 [1.00, 17.10]	0.8960	-	0.2852	-	0.4504	-
		Female H	14.40 [1.10, 16.70]	0.4264	0.2852	-	-	-	0.5982
		Male L	6.65 [3.20, 17.10]	0.1072	-	-	-	0.2975	0.6460
		Male N	4.70 [1.20, 18.50]	-	0.4504	-	0.2975	-	0.7711
		Male H	10.10 [0.90, 17.90]	-	-	0.5982	0.6460	0.7711	-
Palatal	between 4 & 5 (mm)	Female L	11.10 [1.50, 13.60]	-	0.9454	0.0504	0.0613	-	-
		Female N	10.70 [1.50, 15.10]	0.9454	-	0.0038 *	-	0.8764	-
		Female H	8.65 [1.90, 11.10]	0.0504	0.0038 *	-	-	-	0.0180 *
		Male L	11.95 [1.70, 15.30]	0.0613	-	-	-	0.0341 *	0.0295 *
		Male N	9.90 [2.40, 13.90]	-	0.8764	-	0.0341 *	-	0.9934
		Male H	10.10 [0.90, 14.30]	-	-	0.0180 *	0.0295 *	0.9934	-
	between 5 & 6 (mm)	Female L	7.30 [1.30, 15.20]	-	0.3927	0.9127	0.2908	-	-
		Female N	4.50 [1.20, 16.00]	0.3927	-	0.4975	-	0.5128	-
		Female H	10.50 [1.20, 14.10]	0.9127	0.4975	-	-	-	0.3404
		Male L	13.05 [1.80, 16.30]	0.2908	-	-	-	0.3312	0.2310
		Male N	10.60 [1.00, 16.30]	-	0.5128	-	0.3312	-	0.5887
		Male H	4.20 [0.90, 18.40]	-	-	0.3404	0.2310	0.5887	-
	between 6 & 7 (mm)	Female L	5.70 [1.40, 17.10]	-	0.7227	0.6938	0.4986	-	-
		Female N	8.60 [1.80, 17.10]	0.7227	-	0.5743	-	0.6949	-
		Female H	14.40 [1.60, 16.70]	0.6938	0.5743	-	-	-	0.5510
		Male L	6.45 [2.80, 17.10]	0.4986	-	-	-	0.5441	0.5672
		Male N	5.50 [1.20, 18.50]	-	0.6949	-	0.5441	-	0.7585
		Male H	9.95 [1.30, 17.90]	-	-	0.5510	0.5672	0.7585	-

**Table 8:** The chance of alveolar bone thickness of less than 5 mm in each location measured in the skeletal class I population. L: low-angle; N: normo-angle; H: high-angle. 4: first premolar, 5: second premolar, 6: first molar, 7: second molar.

% of <5 mm	5 mm Height						8 mm Height						10 mm Height					
	4–5		5–6		6–7		4–5		5–6		6–7		4–5		5–6		6–7	
	B	P	B	P	B	P	B	P	B	P	B	P	B	P	B	P	B	P
Female L	0.00	0.00	9.52	9.52	0.00	0.00	0.00	0.00	28.57	23.81	28.57	4.76	28.57	23.81	57.14	28.57	52.38	42.86
Female N	0.00	0.00	0.00	0.00	0.00	0.00	0.00	0.00	23.81	23.81	23.81	19.05	14.29	9.52	66.67	57.14	52.38	42.86
Female H	0.00	0.00	5.00	5.00	0.00	0.00	0.00	5.00	20.00	15.00	20.00	15.00	15.00	15.00	40.00	35.00	40.00	35.00
Male L	0.00	0.00	0.00	0.00	0.00	0.00	0.00	0.00	22.22	16.67	0.00	11.11	5.56	5.56	38.89	38.89	27.78	33.33
Male N	0.00	0.00	4.76	4.76	4.76	4.76	9.52	9.52	23.81	19.05	28.57	28.57	14.29	14.29	47.62	47.62	57.14	47.62
Male H	0.00	0.00	0.00	0.00	14.29	14.29	14.29	14.29	28.57	21.43	21.43	21.43	14.29	14.29	64.29	57.14	42.86	42.86

## Data Availability

The data presented in this study are contained within this article.

## References

[1] Baker E.W., Schuenke M., Schulte E. (2011). Head and Neck Anatomy for Dental Medicine.

[2] Iwanaga J., Wilson C., Lachkar S., Tomaszewski K.A., Walocha J.A., Tubbs R.S. (2019). Clinical anatomy of the maxillary sinus: Application to sinus floor augmentation. Anat. Cell Biol..

[3] Shrestha B., Shrestha R., Lin T., Lu Y., Lu H., Mai Z., Chen L., Chen Z., Ai H. (2021). Evaluation of maxillary sinus volume in different craniofacial patterns: A CBCT study. Oral Radiol..

[4] Horiuchi A., Hotokezaka H., Kobayashi K. (1998). Correlation between cortical plate proximity and apical root resorption. Am. J. Orthod. Dentofac. Orthop..

[5] Park J.H. (2020). Temporary Anchorage Devices in Clinical Orthodontics.

[6] Syverson A., Li C., Zheng Z., Proskurnin E., Chung C.H., Zou M. (2022). Maxillary sinus dimensions in skeletal class II population with different vertical skeletal patterns. Clin. Oral Investig..

[7] Maspero C., Farronato M., Bellincioni F., Annibale A., Machetti J., Abate A., Cavagnetto D. (2020). Three-Dimensional Evaluation of Maxillary Sinus Changes in Growing Subjects: A Retrospective Cross-Sectional Study. Materials.

[8] Aktuna Belgin C., Colak M., Adiguzel O., Akkus Z., Orhan K. (2019). Three-dimensional evaluation of maxillary sinus volume in different age and sex groups using CBCT. Eur. Arch. Otorhinolaryngol..

[9] Endo T., Abe R., Kuroki H., Kojima K., Oka K., Shimooka S. (2010). Cephalometric evaluation of maxillary sinus sizes in different malocclusion classes. Odontology.

[10] Ryu J., Choi S.H., Cha J.Y., Lee K.J., Hwang C.J. (2016). Retrospective study of maxillary sinus dimensions and pneumatization in adult patients with an anterior open bite. Am. J. Orthod. Dentofac. Orthop..

[11] Göymen M., Büyüknacar G.B., Güleç A. (2019). Effect of Vertical Growth Pattern on Maxillary and Frontal Sinus Sizes. Eur. J. Ther..

[12] Kosumarl W., Patanaporn V., Jotikasthira D., Janhom A. (2017). Distances from the root apices of posterior teeth to the maxillary sinus and mandibular canal in patients with skeletal open bite: A cone-beam computed tomography study. Imaging Sci. Dent..

[13] Oksayan R., Sokucu O., Yesildal S. (2017). Evaluation of maxillary sinus volume and dimensions in different vertical face growth patterns: A study of cone-beam computed tomography. Acta Odontol. Scand..

[14] Kadioglu O., Currier G.F. (2019). Craniofacial 3D Imaging: Current Concepts in Orthodontics and Oral and Maxillofacial Surgery.

[15] Cao L., Li J., Yang C., Hu B., Zhang X., Sun J. (2019). High-efficiency treatment with the use of traditional anchorage control for a patient with Class II malocclusion and severe overjet. Am. J. Orthod. Dentofac. Orthop..

[16] Li X., Wang H., Li S., Bai Y. (2019). Treatment of a Class II Division 1 malocclusion with the combination of a myofunctional trainer and fixed appliances. Am. J. Orthod. Dentofac. Orthop..

[17] Song G., Chen H., Xu T. (2018). Nonsurgical treatment of Brodie bite assisted by 3-dimensional planning and assessment. Am. J. Orthod. Dentofac. Orthop..

[18] Sawilowsky S.S. (2009). New effect size rules of thumb. J. Mod. Appl. Stat. Methods.

[19] Kau C.H., English J.D., Muller-Delgardo M.G., Hamid H., Ellis R.K., Winklemann S. (2010). Retrospective cone-beam computed tomography evaluation of temporary anchorage devices. Am. J. Orthod. Dentofac. Orthop..

[20] Oktay H. (1992). The study of the maxillary sinus areas in different orthodontic malocclusions. Am. J. Orthod. Dentofac. Orthop..

[21] Jun B.C., Song S.W., Park C.S., Lee D.H., Cho K.J., Cho J.H. (2005). The analysis of maxillary sinus aeration according to aging process; volume assessment by 3-dimensional reconstruction by high-resolutional CT scanning. Otolaryngol. Head Neck Surg..

[22] Velasco-Torres M., Padial-Molina M., Avila-Ortiz G., Garcia-Delgado R., O’Valle F., Catena A., Galindo-Moreno P. (2017). Maxillary Sinus Dimensions Decrease as Age and Tooth Loss Increase. Implant Dent..

[23] Fernandes C.L. (2004). Forensic ethnic identification of crania: The role of the maxillary sinus--a new approach. Am. J. Forensic Med. Pathol..

